# Investigations on the morpho-anatomy and histochemistry of the European mistletoe: *Viscum album* L. subsp. *album*

**DOI:** 10.1038/s41598-023-29799-z

**Published:** 2023-03-21

**Authors:** Valter Paes de Almeida, Irailson Thierry Monchak, João Vitor da Costa Batista, Mirio Grazi, Hartmut Ramm, Vijayasankar Raman, Stephan Baumgartner, Carla Holandino, Jane Manfron

**Affiliations:** 1 Postgraduate Program in Pharmaceutical Sciences, State University of Ponta Grossa, Ponta Grossa, PR Brazil; 2 Society for Cancer Research, Hiscia Institute, 4144 Arlesheim, Switzerland; 3 Department of Pharmaceutical Sciences, Division of Pharmaceutical Technology, University of Basel, 4056 Basel, Switzerland; 4 National Center for Natural Products Research, School of Pharmacy, The University of Mississippi, University, MS USA; 5 Institute of Integrative Medicine, University of Witten/Herdecke, 58313 Herdecke, Germany; 6 Institute of Complementary and Integrative Medicine, University of Bern, 3010 Bern, Switzerland; 7 Laboratório Multidisciplinar de Ciências Farmacêuticas, Faculdade de Farmácia, Universidade Federal do Rio de Janeiro, Rio de Janeiro, 21941-902 Brazil

**Keywords:** Plant sciences, Microscopy, Electron microscopy

## Abstract

*Viscum album* L. (Santalaceae) is an important medicinal plant traditionally used to treat several diseases, including cancer therapy. This paper provides detailed morpho-anatomical characteristics of the leaves, stems and berries of *Viscum album* subsp. *album* growing as hemi-parasite on the branches of *Malus domestica* (Suckow) Borkh. (Rosaceae) to aid species identification and botanical characterization. Additionally, for the first time, microchemical analyses of all tissues and Energy Dispersive X-Ray Spectroscopy analyses of the calcium oxalate crystals are provided for the first time. The plant features leathery presents green leaves with parallel veins, small yellow unisexual flowers in 3-flowered cymes, and the dioecious inflorescences usually consist of three flowers, with female flowers generating white fleshy berries, in which a seed is embedded in the mucilaginous mesocarp, normally containing two embryos. Anatomically, the analyzed leaves were isobilateral and amphistomatic, and showed straight anticlinal epidermal cell walls, thick cuticles with epicuticular wax crystalloids, and paracytic stomata. The midrib is flat on both sides and has a single vascular bundle, whereas the strongly shortened petiole is concave-convex in shape and contains five bundles. The stems show a primary structure with a ring of nine vascular bundles enclosing the pith. Calcium oxalate druses and cubic and quadrangular prisms were observed in different plant parts. The results of this study provide new microscopy information that can help in the authentication of mistletoe raw materials.

## Introduction

The genus *Viscum* (Santalaceae) comprises about 110–141 species^1,2^. Nearly two-thirds of the species are found in Africa and Madagascar, and some in tropical Asia. A few species belonging to the *Viscum album* group have adapted to more temperate regions in Eurasia^3^, of which only two species, *Viscum cruciatum* ex Boiss. and *Viscum album* L., are found in Europe^4^. *Viscum* species, commonly called mistletoes, are shrubby hemi-parasites growing on the aerial parts of host trees and shrubs. They embed into the host branches with a haustorium to nourish and obtain water^5^. These haustoria can extend over more than 5 cm within the host^6^. *Viscum album* grows on more than 450 different species of hosts through its three subspecies, *V. album* subsp. *album*, *V. album* subsp. *austriacum* (Wiesb.) Vollm., and *V. album* subsp. *abietis* (Wiesb.) Abrom., with distinct host preferences and, to some extent, specific geographic distribution^7^.

*Viscum album* subsp. *album* is commonly known as European mistletoe, gui, Mistel, vischio, or muérdago^4^. Its berries consist of a whitish exocarp, a thick mucilaginous mesocarp, a thin endocarp, and a seed that normally contains two embryos^8^. The dispersal of mistletoe seeds depends on birds that feed from the berries during winter: mistle thrush (*Turdus viscivorus*) and waxwing (*Bombicylla garrulus*) gulp several fruits and after digestion excrete the seeds together with the exocarp and the sticky inner mesocarp. The blackcap (*Sylvia atricapilla*) digests only the exocarp and the outer mesocarp, after separating the seed and sticking it with the attached inner mesocarp on a thin branch. Separating seed and exocarp, the birds enable *Viscum album* embryos to germinate^9^.

In spring, the hypocotyl of the embryos elongates, turns to the bark of the host branch and flattens to a holdfast from which the primary haustorium grows with a tip meristem through the bark. When the meristem is embedded into the cambium of the host, it turns into an intercalary meristem, from which synchronously with the secondary thickness growth of the host’s xylem the formation of the primary sinker starts. Originating from the primary haustorium, the cortical strands grow longitudinally and circularly within the inner bark. Whenever their meristems encounter the cambium, they give rise to the formation of secondary sinkers^5,10^.

*Viscum album* is an important medicinal plant. Steiner and Wegman introduced its use in complementary oncological therapy in the early 1900s^11^; since then, many reports have been published describing its medicinal properties^12,13^. In addition to its use in carcinosis treatment, mistletoe is used to treat spleen diseases, menstruation problems, infertility, cardiovascular diseases, ulcers and epilepsy^13–15^. The plant is reported to contain several pharmacologically active compounds, such as amino acids, flavonoids, phenolic acids, polysaccharides, terpenoids, viscotoxins and mistletoe lectins^16–21^.

As only limited information on the morpho-anatomy, micromorphology or histochemistry of *Viscum album* subsp. *album* growing on *Malus domestica* (Suckow) Borkh. is available so far, the present study aimed to fill this gap. This study can also help in taxonomy, species identification, future comparisons with plants growing on other hosts, and quality control of the botanicals.

## Results and discussion

### Important morphological aspects of *V. album* subsp. *album*

**Figure 1:**
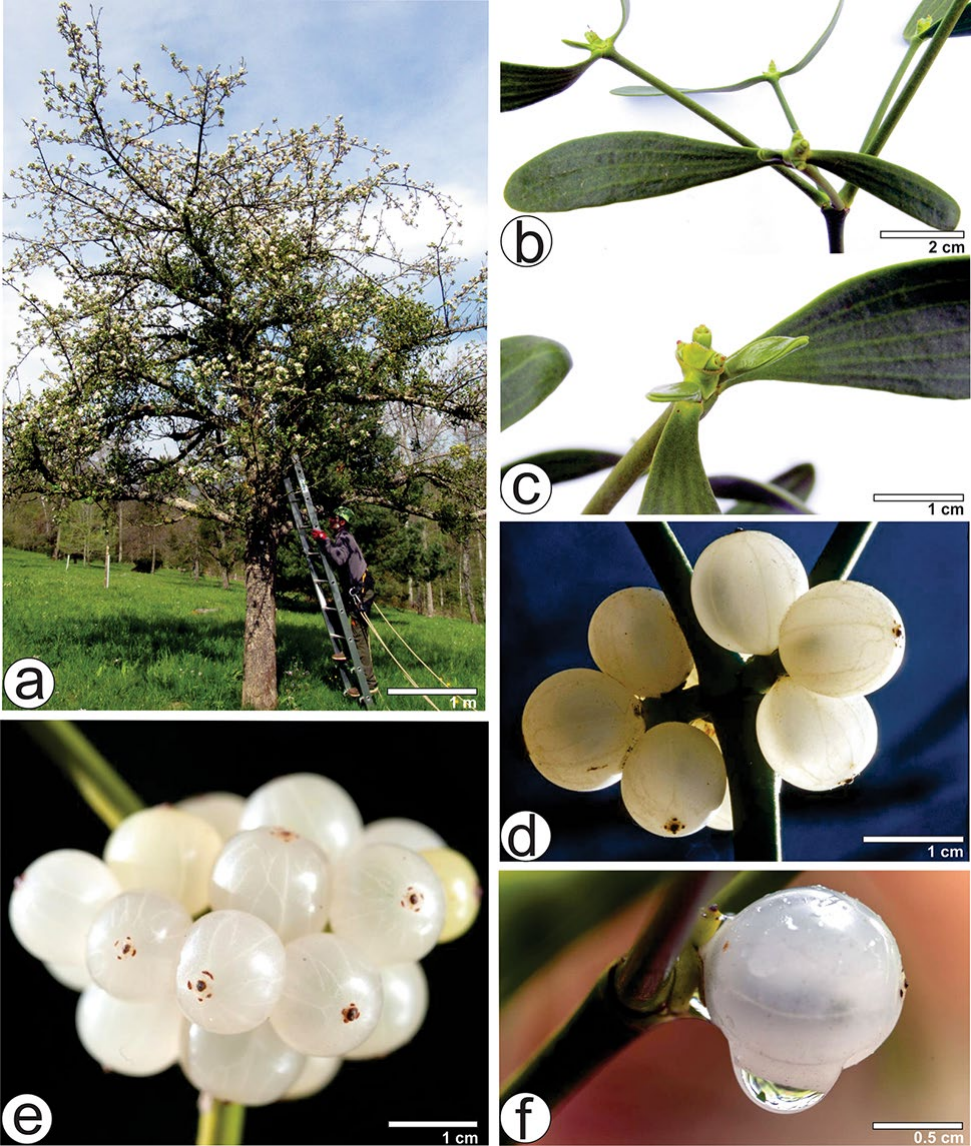
Morphology of *Viscum album* subsp. *album*. (**a**) Plants growing on host branches of *Malus domestica*. (**b**) Several shoots originate from the same node. (**c**) Stem with two opposite leaves. (**d**) Berries. (**e**) Infructescence with berries showing terminal ring of four lateral (tepalar) and one central (stigmal) scars. (**f**) Berry with a raindrop.

*Viscum album* subsp. *album* is a hemi-parasite growing on the branches of deciduous trees such as *Malus domestica*. (Fig. 1a). It grows as dichasium with several articulated, glabrous and green stems (Fig. 1b,c). The leaves are simple, opposite and leathery with an obtuse apex, attenuated base and entire margins, with 3–5 parallel veins, green to yellowish-green. The leaves are highly variable in shape and size, ranging from oblanceolate to obovate-oblong and measuring 2–6 cm long and 0.3–2 cm wide (Fig. 1b,c). Shape and size of leaves may vary considerably, not only within an individual, but between different individuals of the same host tree or of different host trees^22,23^. The fruit is a pseudo berry, globular, whitish, translucent, sessile, 0.8–1 cm in diameter (Fig. 1d–f), crowned by the remains of the dried stigma and remains of the perigone leaves (Fig. 1e); they contain a seed coated by a skinny endocarp and embedded in a very viscous translucent mesocarp. The berries ripen during autumn before winter solstice, with the exocarp changing from green to white or yellowish-white when ripe. Berry color supports the differentiation of *V. album* (European mistletoe) from the closely related *Viscum coloratum* (Kom.) Nakai (Korean mistletoe, with red or yellow fruits) of Eastern Asia^22^.

### Anatomic features by microscopy tools

#### Leaf anatomy

**Figure 2:**
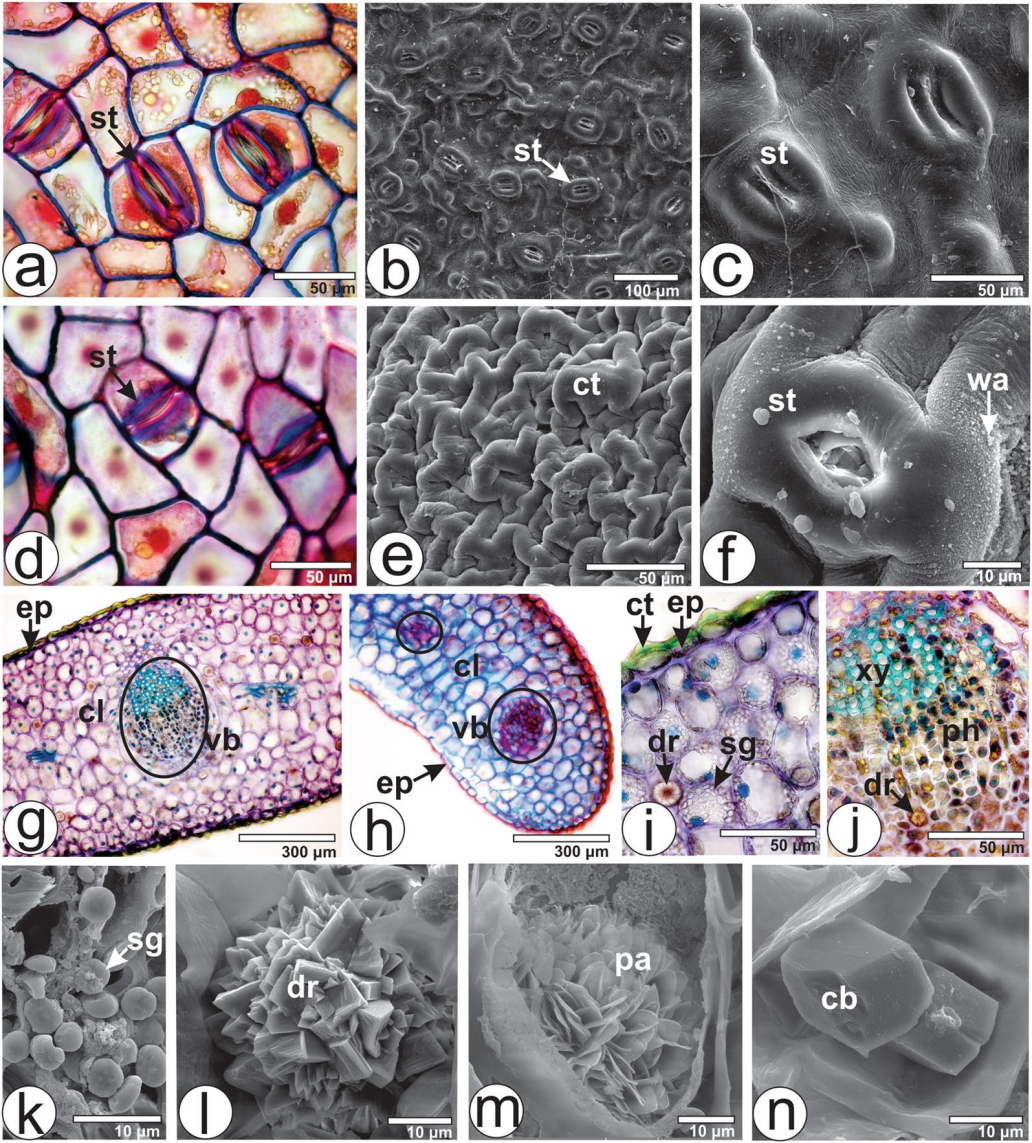
Anatomy of the leaves of *Viscum album* subsp. *album*: (**a**,**d**,**g–j**) light microscopy and (**b**,**c**,**e**,**f**,**k**–**n**) SEM. (**a**–**c**) Adaxial and (**d**–**f**) abaxial sides on the leaf. (**a**–**f**) Leaf in surface view. (**g**–**n**) Transverse sections. *cb* cubic-shaped crystal, *cl* chlorenchyma, *ct* cuticle, *dr* druse, *ep* epidermis, *ph* phloem, *pa* platy aggregation cluster crystal, *sg* starch grain, *st* stomata, *vb* vascular bundle, *wa* epicuticular wax, *xy* xylem.

The surface view of the clarified leaf evidences straight and thin anticlinal cell walls on both adaxial and abaxial epidermises (Fig. 2a,d), covered by a thick cuticle (Fig. 2e). Paracytic stomata (Fig. 2a,d) are located at the same level as the adjacent epidermal cells, and are found on both sides of the lamina (Fig. 2a–d,f). Epicuticular wax crystalloids were observed on both epidermises (Fig. 2f).

Anatomical features such as epidermis with straight anticlinal cell walls, amphistomatic leaves, the presence of paracytic stomata, epicuticular wax crystalloids and thick cuticle, are commonly observed in *Viscum* species^24–30^. However, anomocytic stomata were evidenced in *V. album* subsp. *austriacum*. This subspecies also evidenced glandular trichomes on the adaxial side of the leaves^44^; this feature was not observed in the present study. The type of stomata and trichomes can be used as anatomical markers to distinguish between different subspecies of *V. album* subsp. *austriacum*.

In cross-section, the leaf epidermis is unilayered with strongly cutinized external cell walls on both sides (Fig. 2e,i). Although the leaves are photosynthetic, the mesophyll is undifferentiated and comprises polygonal cells (Fig. 2g,h). However, a dorsiventral mesophyll was found in *V. album* subsp. *album* collected from a host plant *Tilia cordata* Mill.^27^ and *V. album* subsp. *austriacum* growing on *Pinus* sp.^30^. Minor collateral vascular bundles are distributed in the mesophyll (Fig. 2h).

Metcalfe and Chalk^24^ stated that the mesophyll of the biennial leaves of *V. album* is formed by isodiametric cells during the first year, yet a single layer of palisade parenchyma develops towards both sides in the second year. From May until August the shoots of *V. album* have both types of leaves: in the first and in the second year of growth, as can be seen in Fig. 1c.

The mesophyll cells have several starch grains (Fig. 2i,k). They are small, rounded or elongated, and found solitarily or in groups. The presence of starch grains has been reported in various species and subspecies of *Viscum*^30^. Also, different morphotypes of crystals are distributed in the mesophyll (Fig. 2i) and in the vascular bundle (Fig. 2j). They are rectangular prisms, druses (Fig. 2l), platy aggregations of cluster crystals (Fig. 2m), and cubic-shaped crystals (Fig. 2n). Under light microscopy, the druses have a central thick and black region surrounded by many polygonal small crystals (Fig. 2i,j). While cubical, prismatic and druse crystals were observed in the mesophyll of *V. album* subsp. *album* in the present study, only druses were reported in *V. album* subsp. *golestanicum*^29^. The presence of crystals is commonly reported in various species and subspecies of *Viscum*^27,28,31^. However, no crystals were reported in a previous study of *V. album* subsp. *album* by Khan et al.^28^. Also, the presence of platy aggregation cluster crystals in *V. album* subsp. *album* is reported here for the first time. The grouping of more than one crystal morphotype can be present as the feature of the subspecies, species, section, subgenus or genus, giving support to the taxonomy^32^.

The midrib is flat on both sides (Fig. 2g). The vascular system is represented by a central collateral vascular bundle (Fig. 2g). Fibers are abutting the xylem and phloem (Fig. 2g,j). Bicollateral vascular bundles were found in *V. album* and in *V. cruciatum* Sieber ex Boiss. in a study by Khan et al.^28^.

**Figure 3:**
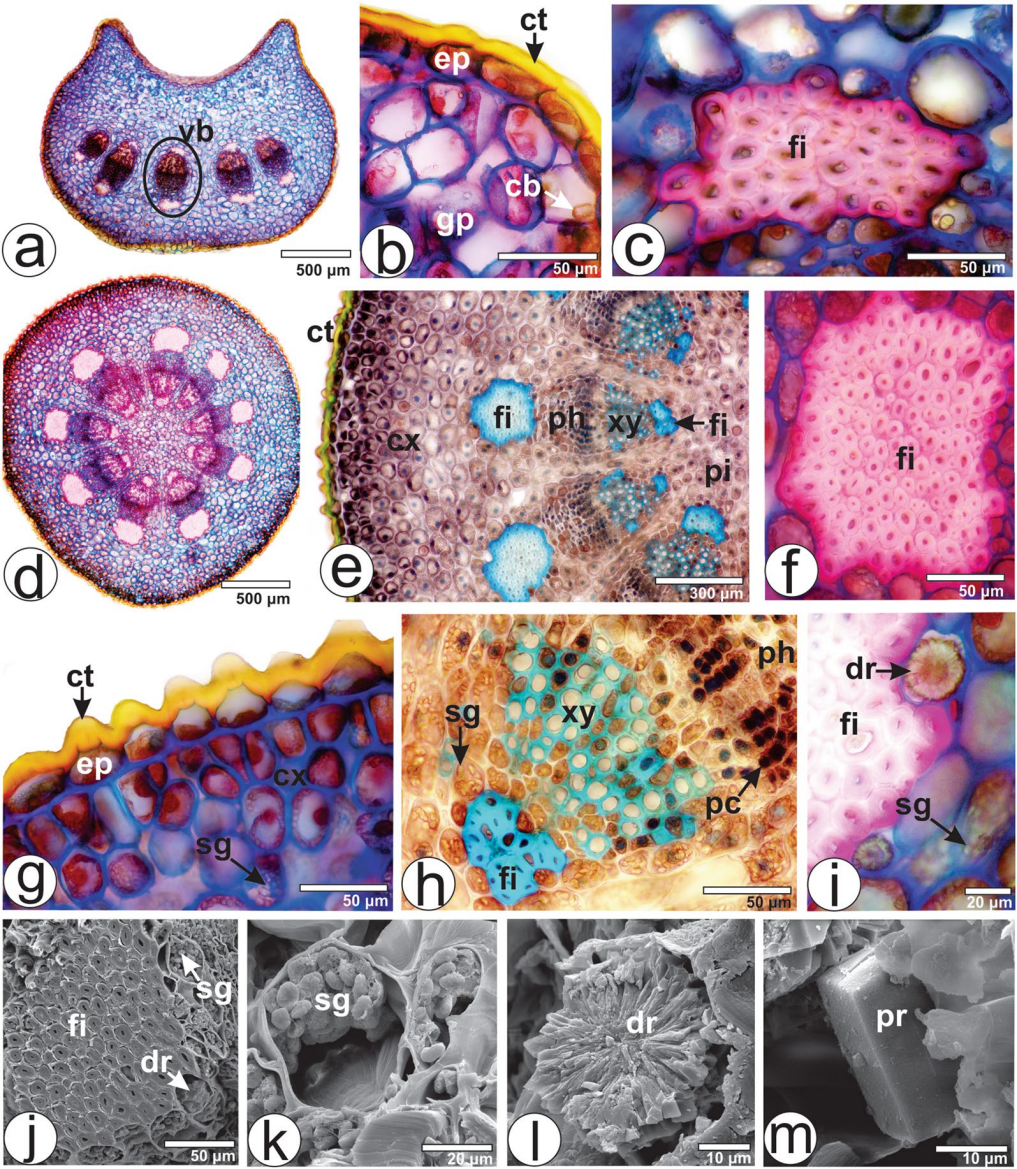
Leaf (petiole) and stem anatomy of *Viscum album* subsp*. album* in cross-section: (**a**–**c**) Petiole. (**d–m**) Stem. (**a**–**i**) light microscopy and (**j**–**m**) SEM. *cb* cubic-shaped crystal, *ct* cuticle, *cx* cortex, *dr* druse, *ep* epidermis, *fi* fiber, *gp* ground parenchyma, *pc* phenolic compounds, *ph* phloem, *pi* pith, *pr* quadrangular prismatic crystal, *sg* starch grain, *vb* vascular bundle, *xy* xylem.

The strongly shortened and weakly differentiated petiole of *V. album* subsp. *album*, sectioned transversely at the medial portion, had a concave-convex shape with two wings on adaxial side (Fig. 3a). The epidermal cells are covered by a thick cuticle (Fig. 3b). The ground tissue is undifferentiated as in the lamina. The vascular system is collateral and was represented by five bundles organized in an open arc (Fig. 3a). A cap of perivascular fiber adjoins the xylem and phloem (Fig. 3c). The same crystal morphotypes previously described for lamina were observed in the ground parenchyma (Fig. 3b). However, Khan et al.^28^ have not reported any crystal in the petiole of *V. album*. Although the outline of the petiole is a general diagnostic feature for higher plants^33^, there are only few studies involving the anatomy of the petiole of *Viscum* species. The same features were found in *V. album* subsp. *album* and *V. album* subsp. *golestanicum*^29^. However, 6–7 vascular bundles were observed in *V. album* and *V. cruciatum* in the study by Khan et al.^28^.

#### Stem anatomy

The stem is circular in cross-section (Fig. 3d). The epidermis is unilayered with tangentially elongated cells (Fig. 3g). The external wall is particularly thick and fully cutinized (Fig. 3g). Over each external wall, in the middle of the cell, a projecting monticule or papilla, half-moon or elliptical shaped, could be observed (Fig. 3e,g). The cortex is discreetly collenchymatous and formed by several layers of parenchyma cells that increase in size towards the vascular system (Fig. 3e). The vascular system is represented by nine vascular bundles forming a ring and delimiting the pith (Fig. 3d). The row and number of cells in the xylem are higher than in the phloem (Fig. 3e). One cap of perivascular fibers is attached to the xylem and another to the phloem (Fig. 3d–f,i), the latter being more developed. The pith is at the center of the stem and is composed of parenchyma cells (Fig. 3d). Idioblasts containing brownish substances corresponding to phenolic compounds are present in the phloem (Fig. 3h). Cortical, vascular and medullary parenchyma are filled with starch grains (Fig. 3g–k) with the same features previously described for leaves; however, these are not present in the first layers of the cortex.

Druses (Fig. 3i,j,l), cubic and quadrangular prismatic (Fig. 3m) crystals are also spread in the stem tissues. Abundant crystals are present in the cortex and phloem of *V. cruciatum*, yet are absent in *V. album*^28^. Mehrvarz et al.^29^ have reported that the distribution and morphotype of the calcium oxalate crystals could provide valuable support in delimitating of subspecific taxa in *V. album*. The use of calcium oxalate crystals in solving taxonomic problems has been suggested in previous studies of other plant groups such as *Baccharis*^32,34^, *Eucalyptus*^35^ and *Piper*^36^.

Most characteristics observed in the present study have been described for *Viscum* species^27–30^. The vascular bundles showed poorly developed phloem but well-developed xylem. It means that they can resourcefully take up water and prepared inorganic and organic nutrients from the host plant^37^.

#### Berry anatomy

**Figure 4:**
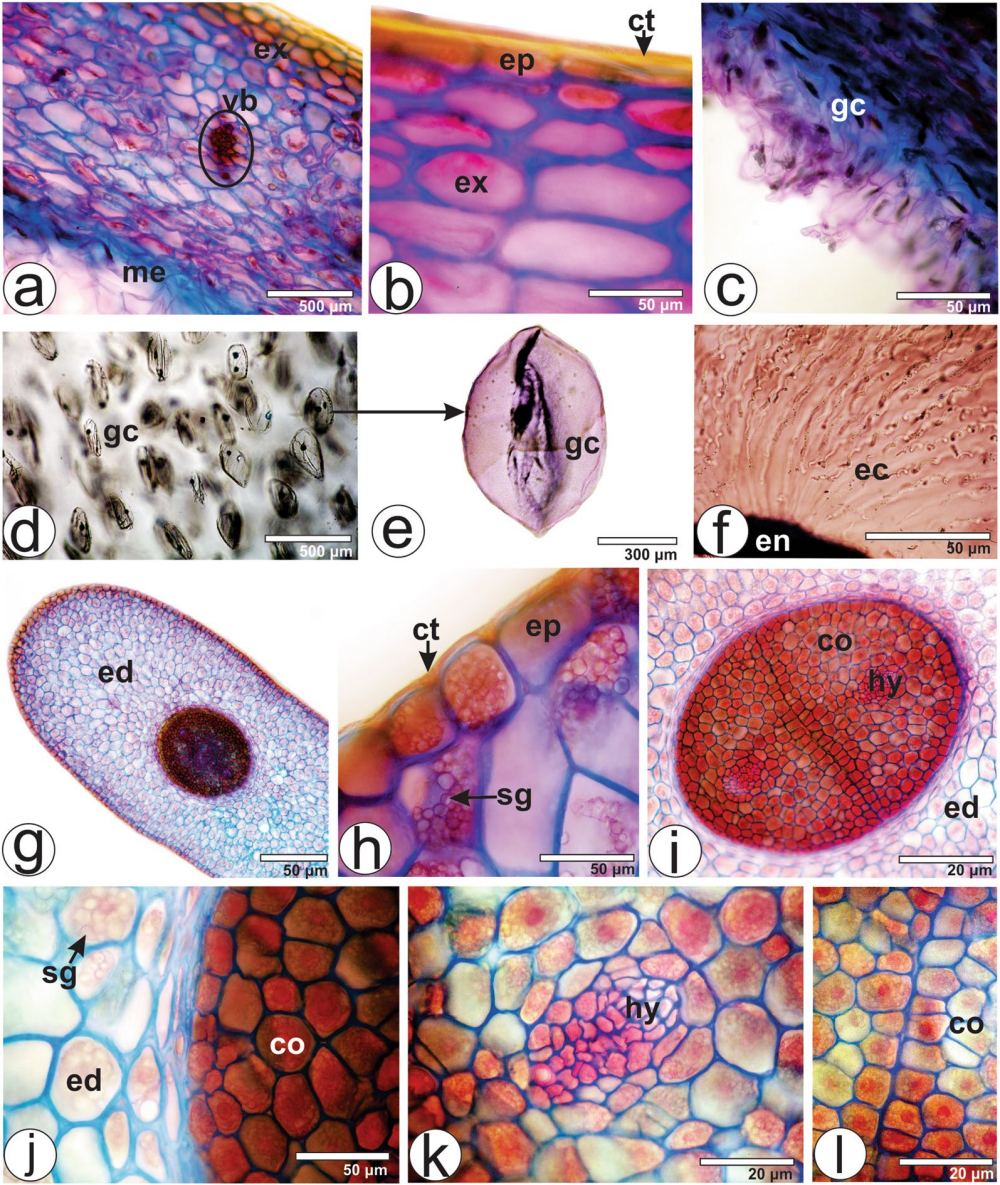
Berry and seed anatomy of *Viscum album* subsp. *album* in cross-section. (**a**–**f**) Berry. (**g**–**l**) Seed. *ct* cuticle, *co* cotyledon, *ec* elongated cells with spiral thickening, *ep* epidermis, *ed* endosperm, *en* endocarp, *ex* exocarp, *gc* globular cells, *hy* hypocotyl, *me* mesocarp, *sg* starch grain, *vb* vascular bundle.

The berry, in frontal view, presents an epidermis with straight anticlinal cell walls. In cross-section, the exocarp is formed by an unilayered epidermis covered by a smooth cuticle and 3–4 layers of angular collenchyma (Fig. 4a,b). In the medial region, small collateral vascular bundles are present (Fig. 4a). The mesocarp, which surrounds the seed, contains an outer fleshy layer and an inner sticky viscin tissue (Fig. 4a,c). The fleshy layer is not sticky.

The viscin tissue is composed of strongly vacuolated globular cells (Fig. 4c–e) and highly viscous, sticky and extensible, long cells with spirally thickened walls located towards the endocarp (Fig. 4f). These cells contain druses and rectangular prism crystals, and a few starch grains. The endocarp is thin and encloses the seed. Grazi and Urech^38^ reported that there is no information on the extensible filaments between the seeds and the outer mesocarp in *V. album* subsp. *abietis* and *V. album* subsp. *austriacum* growing on conifers.

#### Seed anatomy

The seed is green and does not have a seed coat. Heide-Jørgensen^39^ stated that in *Viscum* genus no seed coat is formed since the integuments are lacking. In cross-section, the seed has an unilayered epidermis covered by a cuticle (Fig. 4h). The epidermal cells have several starch grains (Fig. 4h). The endosperm cells are large, parenchymatous and contain chlorophyll and large amounts of starch grains (Fig. 4g–j). The seed usually has two embryos, each with two cotyledons and a hypocotyl (Fig. 4i–l). The number of embryos per berry varies in *Viscum album*. Commonly, *V. album* subsp. *album* has two embryos, sometimes only one embryo, and rarely three or even four embryos, while the percentage of monoembryonal berries is higher in subsp. *abietis* and *V. album* subsp. *austriacum*^8,26^.

### Elemental analysis of crystals using EDS

**Figure 5:**
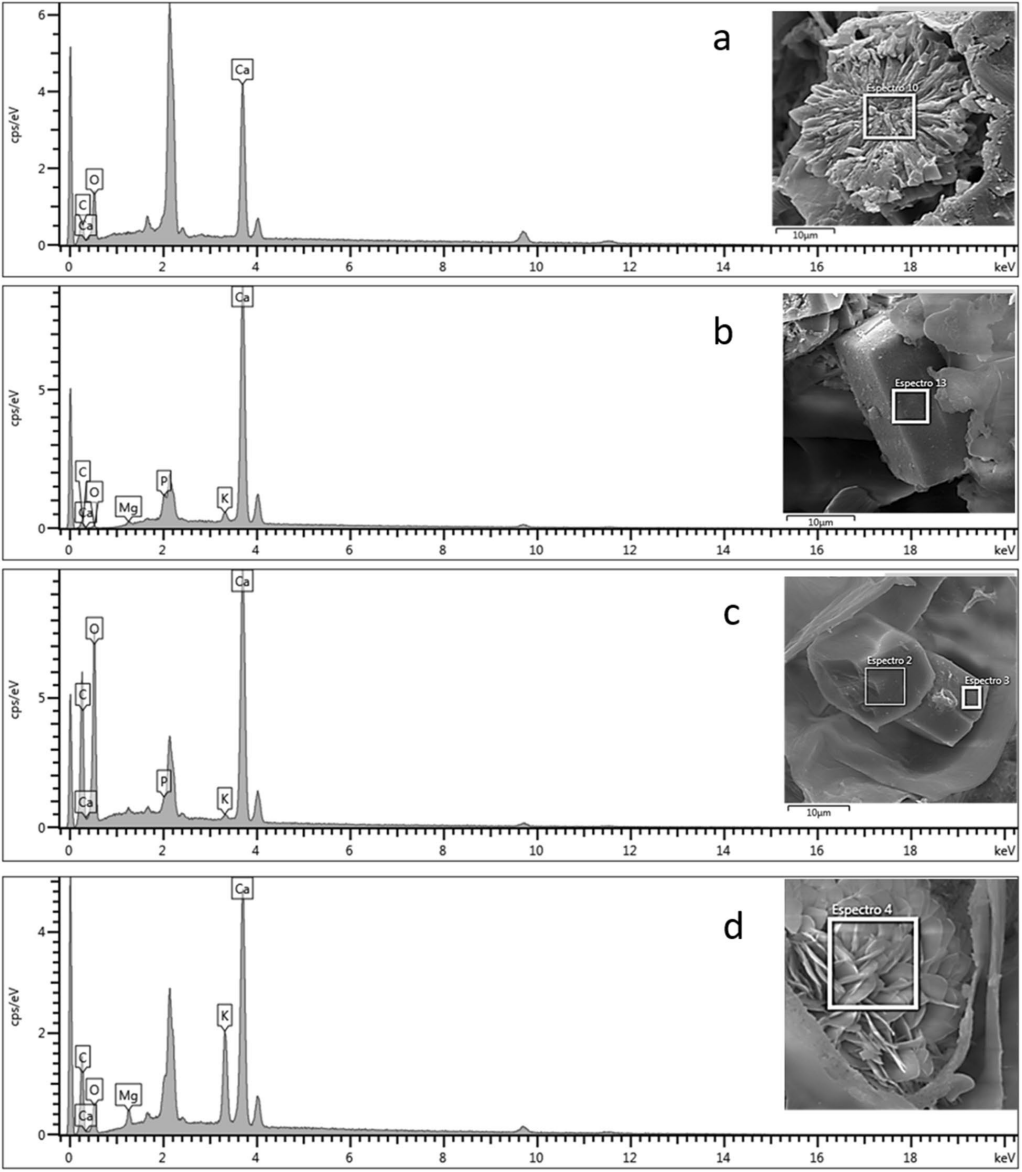
SEM image and EDS spectra of the druse (**a**), rectangular prism (**b**), cubic prism (**c**) and platy aggregated (**d**) crystals from *Viscum album* subsp. *album*. The prominent unlabeled peak at 0 keV is the noise peak, and the peak near 2.1 keV is for gold (Au) used for sputter-coating the samples for SEM analysis.

The chemical composition of the crystals occurring in plants can be identified using EDS^40,41^. In the present study, four morphotypes of calcium oxalate crystals were found in various tissues of *V. album* subsp. *album* (Figs. 2i,j,l–n, 3b,i,j,l,m). The EDS spectra of druses (Fig. 5a) exhibited prominent peaks for calcium (Ca), carbon (C), and oxygen (O). However, in addition, other elements such as magnesium (Mg), phosphorous (P), and potassium (K) were also found in minor concentrations in the cubic (Fig. 5c) and rectangular prisms (Fig. 5b), and platy aggregation crystals (Fig. 5d). The most common minerals formed by plants are calcium oxalate, calcium carbonate and silica^42^. This is the first study of the elemental chemical composition of crystals of *Viscum* species using EDS.

### Histochemical tests: a link between chemical compounds and its anatomical compartmentalization

Microchemical analyses using specific reagents and stains under light microscopy are helpful for the characterization of chemical compounds present in plant tissues. They can also be used to detect, within specific cells and tissues, the distribution and accumulation of the compounds or groups of secondary metabolites, such as lipophilic material, protein, mucilage, lignified elements, and phenolic compounds^43–53^. In the present study, an array of histochemical tests were conducted using several (Table 1). No histochemical tests were previously reported for *V. album* subsp. *album*.

**Table 1:** Results of microchemical tests with *V. album* subsp. *album*.

Microchemical reagents	Reaction	Occurrence in plant organs					
		Leaf			Stem	Berry	Seed
		Lamina	Midrib	Petiole			
Sudan III	Staining lipids red or red–orange	Cuticle and oil bodies (epidermis and mesophyll)	Cuticle and oil bodies (epidermis and ground parenchyma)		Cuticle	Cuticle and oil bodies in the exocarp cells	Cuticle
Sudan black	Staining lipids black						
Nile blue	Stains neutral fats in red and fatty acids in blue	Oil bodies	Oil bodies		ND	Oil bodies	ND
Potassium dichromate	Gives phenolics a brown or reddish-brown color	Minor vascular bundles	Vascular bundles		Cortex and vascular bundles	ND	
Ferric chloride	Turns phenolics to dark brown						
Vanillin/HCl	Gives rise to a bright red vanillin-tannin condensate	ND					
Phloroglucinol/HCl	Reveals lignified elements in pink to red color	Fibers and vessel elements			Fibers and vessel elements	Elongate cells	Endocarp cells
PAS (periodic acid-Schiff)	Neutral polysaccharides become magenta	Epidermis and mesophyll cells	Epidermis, ground parenchyma and phloem		Phloem and pith	Exocarp and mesocarp	All the cell walls, especially near the embryo
Iodine solution	Stain starch in dark blue to black	Epidermis and mesophyll cells	Ground parenchyma	ND	Epidermis, cortex and pith	Mesocarp (globular cells region)	Endosperm
Xylidine Ponceau	Reveals protein bodies in red color	Protein globular corpuscles					Endosperm, hypocotyl and embryo
Coomassie Brilliant Blue	Turns protein bodies blue	Protein globular corpuscles					
Ruthenium red	Reacts with pectins, mucilages and gums turning them pink to red	Epidermis, phloem and mesophyll cells	Epidermis, ground parenchyma and phloem			Epidermis and exocarp cells	
Dragendorff, Ellram and Wagner	Gives alkaloids an orange to reddish-brown color	ND					

*ND* not detected.

**Figure 6:**
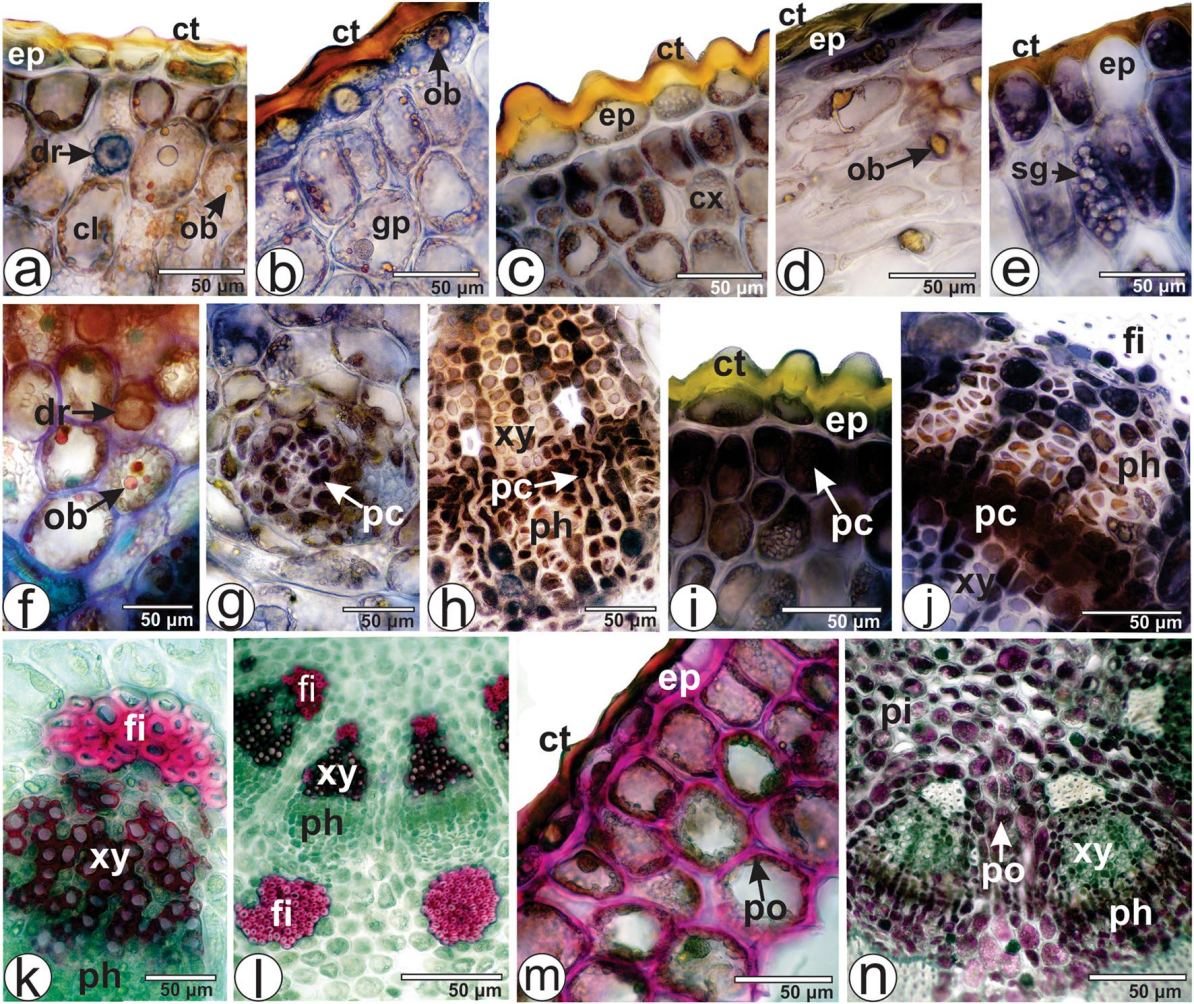
Microchemical tests of *Viscum album* subsp. *album* in cross-section. (**a**,**b**,**g**,**h**,**k**,**m**) Leaf [(**a**,**g**) Lamina, (**f**,**m**,**k**) Midrib, (**b**,**h**) Petiole]. (**c**,**i**,**j**,**l**,**n**) Stem. (**d**) Berry. (**e**) Seed. Reagents: (**a**–**e**) Sudan III. (**f**) Nile blue. (**g**,**h**) Potassium dichromate. (**i**,**j**) Ferric chloride. (**k**,**l**) Phloroglucinol/HCl. (**m**,**n**) PAS (periodic acid-Schiff reagent). *cl* chlorenchyma, *ct* cuticle, *cx* cortex, *dr* druses, *ep* epidermis, *fi* fibers, *gp* ground parenchyma, *hy* hypocotyl, *ob* oil bodies, *pc* phenolic compounds, *ph* phloem, *po* neutral polysaccharides, *sg* starch grain, *xy* xylem.

*Viscum album* subsp. *album* reacts positively with Sudan III and Sudan black, showing lipophilic compounds in cuticles of the leaves [lamina (Fig. 6a) and petiole (Fig. 6b)], stems (Fig. 6c), berries (Fig. 6d) and seeds (Fig. 6e). Oil bodies also react with this reagent^32^ and are found in the epidermis and mesophyll (Fig. 6a) of the lamina, in the epidermis (Fig. 6b) and ground parenchyma (Fig. 6b) of the petiole, and in the exocarp cells of berries (Fig. 6d). Oil bodies (Fig. 6f) stain with Nile blue as well, indicating that the bodies contain neutral fats. Nile blue is a basic dye in the oxazine group that stains neutral fats and fatty acids in red and blue, respectively^54^.

Phenolic compounds can be evidenced using different reagents: ferric chloride solution, potassium dichromate solution and vanillin-hydrochloric. *Viscum album* subsp. *album* reacts positively with potassium dichromate (Fig. 6g,h) and ferric chloride (Fig. 6i,j) solutions, and the cells containing phenolic compounds were found in the vascular bundles of leaves and stems. However, no condensed tannins were found in this study.

Lignified elements can be detected using phloroglucinol/HCl. Lignin is a compound present in several or all the secondary wall layers that contribute to the lignification process resulting in the modification of cell wall properties^35^. In the present study, lignified elements were evidenced in fibers and vessel elements in the leaves and stems (Fig. 6k,l).

Plant polysaccharides are macromolecules comprised of several identical or different monosaccharides with *α*- or *β*-glycosidic bonds. In microchemical tests, the Schiff reagent is frequently used to distinguish certain mucins and other carbohydrates in a staining sequence called the PAS (periodic acid-Schiff) test. Polysaccharides that comprehend a pair of adjacent hydroxyl groups can be oxidized to aldehydes by periodic acid. The aldehydes react with colorless Schiff reagent and the positive tissue sites become magenta. Neutral polysaccharides are spread in the cell walls in the leaf epidermis, midrib ground parenchyma (Fig. 6m), and phloem and pith in the stem (Fig. 6n), exocarp and mesocarp of berry and all the cell walls in the seed, especially near the embryo.

**Figure 7:**
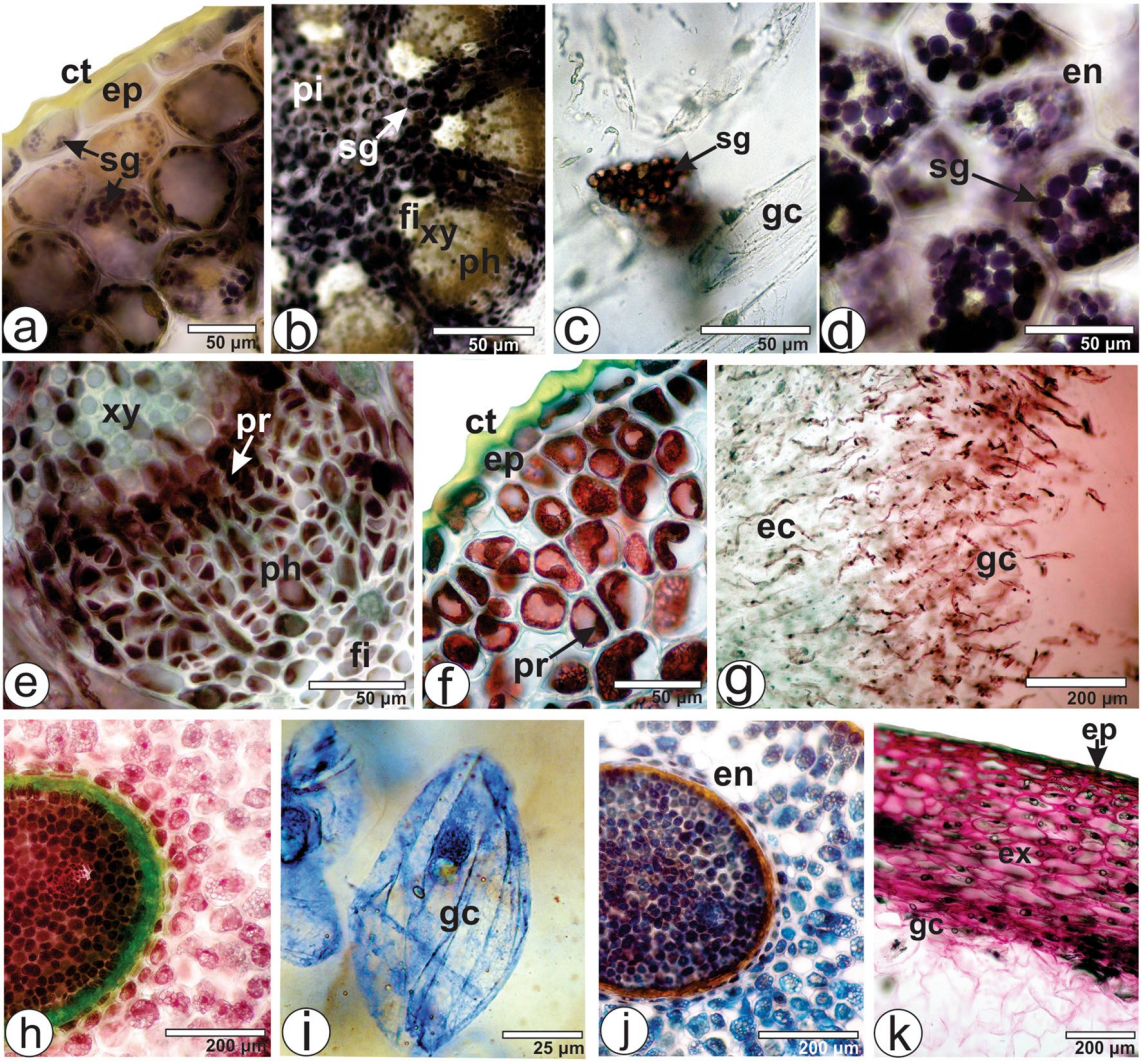
Microchemical tests of *Viscum album* subsp. *album* (Cross-sections of (**a**) Lamina, (**e**) Midrib, (**b**,**f**) Stem, (**c**,**g**,**i**) Berry, and (**d**,**j**,**k**) Seed. Reagents: (**a**-**d**) Iodine solution. (**e**–**h**) Xylidine Ponceau. (**i**,**j**) Coomassie Brilliant blue. (**k**) Ruthenium red. *ct* cuticle, *ec* palisade-like elongated cells with spirally thickened walls, *en* endosperm, *ep* epidermis, *ex* exocarp, *fi* fiber, *gc* globular cells, *ph* phloem, *pi* pith, *pr* protein, *sg* starch grain, *xy* xylem.

Iodine solution is used to identify stain starch. Almost all other structures stain yellow, but this color has no specific meaning. Starch is one of the main ergastic substances of the protoplast and contains a long chain of polysaccharides grouped around a hilum and forming characteristic granules. Starch grains are found in epidermis and mesophyll cells (Fig. 7a) of the lamina and ground parenchyma of the midrib. They are widespread in the cortex, medullary rays, and pith of the stem (Fig. 7b), in some mesocarp cells in the berry (Fig. 7c), and in the endosperm of the seed (Fig. 7d). They are small, rounded and found in groups.

Xylidine Ponceau and Coomassie Brilliant Blue react positively with *V. album* subsp. *album.* The presence of protein globular corpuscles is observed occasionally in the leaves (Fig. 7e), stems (Fig. 7f) and berries (Fig. 7g,i). However, they are commonly found in seeds, especially in the endosperm (Fig. 7h,j). The protein globular corpuscles are vacuolated structures that accumulate reserve protein in the seeds and are called protein bodies or aleurone grains.

Ruthenium red is a polycationic stain that reacts with pectic substances, mucilage, and gums^56^. These substances are detected in almost all cells in the leaves, stems, berries (Fig. 7k) and seeds, yet not in fibers and xylem elements. Azuma et al.^57^ analyzed the cellulose system of the viscous fibrous cellulosic polysaccharide (viscan) in the viscin tissue of *V. album* and reported that it is formed by cellulose and hemicellulose together with a minor amount of pectic substance. The viscin tissue assists in attaching the mistletoe berries to the host branches.

Dragendorff, Ellram and Wagner are reagents that detect alkaloids^44,45^. These secondary metabolites were not detected in any of the organs of the *V. album* subsp. *album*.

## Materials and methods

### Plant materials

The use of plants in the present study complies with international institutional guidelines. *Viscum album* subsp. *album* was harvested from *Malus domestica* growing on a cultivated natural site in Switzerland (belonging to the Society for Cancer Research-VfK). Therefore, no permission for harvesting was needed. The plants were identified by Dr. Marcelo Guerra Santes (Universidade Estadual do Rio de Janeiro, Brazil) and a voucher specimen (C.H. Quaresma 18.332) was deposited at the Herbarium of the Faculdade de Formação de Professores, Universidade Estadual do Rio de Janeiro, Brazil. Fresh specimens of the leaves, stems, berries and seeds of *V. album* subsp. *album* were collected for the first investigations in July 2016 from plants growing on the branches of *Malus domestica* from the Basel area (Rüti, Himmelried) in Switzerland. To develop the present study, the leaves and stems, as well as the berries and seeds of *V. album* subsp. *album* were collected in April 2021 and February 2022, respectively, on the same location in Switzerland (Rüti, Himmelried).

### Preparation of samples for light microscopy

The mistletoe bush used in this study was about seven years old. The following samples were harvested from the same bush: six young leaves (1-year-old), four young stems (1st year), six berries, six matured leaves (2 years old), and four matured stems (2 years old). Only perfect and healthy organs, without diseases or infections, were collected following methodology previously standardized by our group^18,19,21^. Fresh samples of the stems, leaves, berries and seeds were collected from the plant and fixed in FAA 70 (formalin, acetic acid, 70% ethanol, 5:5:90 v/v/v) for three days. The samples were then washed with water, transferred into 70% ethanol, and free-hand sections were prepared using razor blades (thickness of cross-sections 15–30 μm). Selected sections were stained in toluidine blue^58^ and in a combination of Astra blue and basic fuchsin^59^. Then the sections were mounted in a drop of glycerin (50%)^60^ on glass slides, covered with a coverslip, and sealed with transparent nail polish. For the analysis of epidermal surfaces, small sections of the leaves were washed and then treated with sodium hypochlorite solution (50%) until translucent^61^. The materials were then washed with distilled water and neutralized in an acetic acid solution (5%). The sections were rewashed with distilled water, stained in safranin^60^ and mounted as described above.

### Histochemical analyses

A series of multiple histochemical tests were conducted using different chemical reagents and stains (Table 1). Free-hand cross-sections of fresh material were exposed to phloroglucinol/HCl to detect traces of lignin^43^. Ellram, Wagner^44^ and Dragendorff^45^ reagents were used to detect alkaloids^44,45^. Potassium dichromate (10%) 46 and ferric chloride (5%) were used to detect the presence of phenolic substances^47^. Hydrochloric vanillin solution (0.5%) was applied to reveal condensed tannins^48,55^. Sudan III and Sudan black B were used to detect lipophilic compounds^49^. In addition, Nile blue sulfate was used to expose neutral and/or acidic lipids^50^; iodine solution (2%) to identify starch grains^47^; ruthenium red solution (0.002%) for pectins^47^; PAS (periodic acid-Schiff reagent) test for polysaccharides^51^; and Xylidine Ponceau^52^ and Coomassie Brilliant blue^53^ were used to evidence proteins. The reaction methods and results of these histochemical tests are summarized in Table 1. Appropriate controls were performed in parallel with the tests. Photomicrographs were captured using an Olympus CX 31 light microscope with the attached C-7070 control unit. The microscopic procedures were conducted in the Laboratory of Pharmacognosy at the State University of Ponta Grossa (UEPG, Brazil).

### Preparation of samples for scanning electron microscopy (SEM)

The FAA-fixed samples were dehydrated by passing through increasing concentrations of ethanol in water (70%, 80%, 90%, and 100%). The samples were then dried in a Balzers CPD 030 critical point dryer (BAL-TEC AG, Balzers, Liechtenstein) supplied with liquid CO_2_ and then coated with gold using a Quorum (model SC7620) sputter coater. Photomicrographs were recorded using a Mira 3 field-emission scanning electron microscope (Tescan, Brno-Kohoutovice, Czech Republic).

### Energy dispersive X-ray spectroscopy (EDS)

During the SEM procedure, EDS was performed to obtain the chemical composition spectra of the crystals. This analysis was made for the crystals as well as the cells devoid of crystals as a control, using an EDS detector on the same variable pressure microscope at 15 kV. SEM and EDS analyses were performed at the Multiuser Laboratory Complex (C-Labmu) of the State University of Ponta Grossa (UEPG, Brazil).

## Conclusion

This study provides valuable anatomical, micromorphological and microchemical information about *Viscum album* subsp. *album*. The anatomy features of the leaf, stem, berry and seed are significant, and this study reveals several attributes of potential taxonomic importance at the genus and species levels. The present study will also provide a basis for future studies involving other taxa in the family for a better understanding of the morpho-anatomy, interaction with host plants, and phylogeny of this interesting group of hemi-parasitic plants.

## Data Availability

The datasets used and/or analyzed during the current study are available from the corresponding author on reasonable request.

## References

[1] POWO. *Plants of The World Online* https://powo.science.kew.org/taxon/urn:lsid:ipni.org:names:30000304-2 (2022).

[2] WFO. *The World Flora Online* http://www.worldfloraonline.org/taxon/wfo-4000040344 (2022).

[3] Kirkup, D. W., Polhill, R. M. & Wiens, D. Viscum in the context of its family, Viscaceae, and its diversity in Africa. In* Mistletoe: The Genus Viscum* (ed. Büssing, A.) 7–29 (CRC Press, 2000).

[4] Becker, H. European mistletoe: Taxonomy, host trees, parts used, physiology. In *Mistletoe: The Genus Viscum* (ed. Büssing, A.) 31–43 (CRC Press, 2000).

[5] Sallé, G. Germination and establishment of *Viscum album* L. In* The Biology of Mistletoes* (eds. Calder, D. M. & Bernhardt, P.) 145–159 (Academic Press, 1983).

[6] Mylo MD (2021). Advances on the visualization of the internal structures of the European Mistletoe: 3D reconstruction using microtomography. Front. Plant. Sci..

[7] Barney CW, Hawksworth FG, Geils BW (1998). Hosts of *Viscum album*. Eur. J. For. Pathol..

[8] Ramm, H., Urech, K., Scheibler, M. & Grazi, G. Cultivation and development of *Viscum album* L. In *Mistletoe: The Genus Viscum* (ed. Büssing, A.) 75–94 (Taylor & Francis, 2004).

[9] Melado A, Zamora R (2014). Generalist birds govern the seed dispersal of a parasitic plant with strong recruitment constraints. Oecologia.

[10] Thoday D (1951). The haustorial system of *Viscum album*. J. Exp. Bot..

[11] Ramm, H. Mistletoe through cultural and medical history: The all-healing proves to be a cancer-specific remedy. In *Mistletoe: From Mythology to Evidence-Based Medicine* (eds. Zanker, K. S. & Kaveri, S. V.) 1–10 (Translational Research in Biomedicine, 2015).

[12] Loef M, Walach H (2020). Quality of life in cancer patients treated with mistletoe: A systematic review and meta-analysis. BMC Complement. Med. Ther..

[13] Ostermann T (2020). A systematic review and meta-analysis on the survival of cancer patients treated with a fermented *Viscum album* L. extract (iscador): An update of findings. Complement. Med. Res..

[14] Büssing, A. *Mistletoe: The Genus Viscum* (ed. Büssing, A.) 1–261. (CRC Press, 2000).

[15] Poruthukaren KJ, Palatty PL, Baliga MS, Suresh S (2014). Clinical evaluation of *Viscum album* mother tincture as an antihypertensive: A pilot study. J. Evid. Based Complement. Altern. Med..

[16] Schaller G, Urech K, Grazi G, Giannattasio M (1998). Viscotoxin composition of the three European subspecies of *Viscum album*. Planta Med..

[17] Urech K, Baumgartner S (2015). Chemical constituents of *Viscum album* L.: Implications for the pharmaceutical preparation of mistletoe. Transl. Res. Biomed..

[18] Melo MNO (2022). *Viscum album* mother tinctures: Harvest conditions and host trees influence the plant metabolome and the glycolytic pathway of breast cancer cells. Front. Pharmacol..

[19] Holandino C (2020). Phytochemical analysis and in vitro anti-proliferative activity of *Viscum album* ethanolic extracts. BMC Complement. Med. Ther..

[20] Peñaloza E (2020). Comprehensive metabolome analysis of fermented aqueous extracts of *Viscum album* L. by liquid chromatography−High resolution tandem mass spectrometry. Molecules.

[21] Jäger T (2021). Metabolomics by UHPLC-Q-TOF reveals host tree-dependent phytochemical variation in *Viscum album* L. Plants..

[22] Zuber D (2004). Biological flora of Central Europe: *Viscum album* L. Flora Morphol. Distrib. Funct. Ecol. Plants..

[23] Bilgili E, Alperen Coşkuner KA, Öztürk M (2020). Leaf area—sapwood area relationship in Scots pine (*Pinus sylvestris* L.) under mistletoe (*Viscum album* ssp. austriacum) infection. Dendrobiology.

[24] Metcalfe, C. R. & Chalk, L. *Anatomy of the Dicotyledons* (Clarendon Press, 1979).

[25] Haas K, Bauer M, Wollenweber E (2003). Cuticular waxes and flavonol aglycones of mistletoes. Z. Naturforsch. C..

[26] Becker, H. European Mistletoe: Taxonomy, host trees, parts used, physiology. In *Mistletoe: The Genus Viscum* (ed. Büssing, A.) 31–43 (Taylor & Francis, 2004).

[27] Andronache A, Toma I, Toma C (2006). The structure of vegetative organs in *Viscum album* and *Loranthus europaeus*. Analele Stiinţ. ale Univ..

[28] Khan MA, Sharif T, Ahmad M, Zafar M, Tareen RB (2009). Anatomical characterization of parasitic plants of Pakistan. Pak. J. Bot..

[29] Mehrvarz SS, Shavvon RS, Golmohammadi N (2012). Notes on the genus *Viscum* (Viscaceae) in Iran: A new combination based on morphological evidence. Afr. J. Agric. Res..

[30] Elkiran O (2022). Comparative anatomical study of *Viscum album* subsp. *album* L. and *Viscum album* subsp. *austriacum* (Wiesb.) Vollman. *Ejons Int*. J. Math. Eng. Nat. Sci..

[31] Mbagwu FN, Unamba CIN, Ezeibekwe KO (2009). Leaf anatomical characteristics of five variants of the genus *Viscum* L. (Loranthaceae). Agric. J..

[32] Almeida VP (2021). Microscopy and histochemistry of leaves and stems of *Baccharis* subgenus *Coridifoliae* (Asteraceae) through LM and SEM-EDS. Microsc. Microanal..

[33] Almeida VP (2017). Comparative morphoanatomical analysis of *Mikania* species. Rev. Bras. Farmacogn..

[34] Manfron, J, Farago, P. V., Khan, I. A. & Raman, V. Morpho-anatomical characteristics of species of Baccharis. In *Baccharis from Evolutionary and Ecological Aspects to Social Uses and Medicinal Applications* (eds. Fernandes, G. W., Oki, Y. & Barbosa, M.) 217–237 (Springer Nature, 2021).

[35] Brito PS (2021). Light and scanning electron microscopy, energy dispersive X-ray spectroscopy, and histochemistry of *Eucalyptus tereticornis*. Microsc. Microanal..

[36] Santos VLP (2021). Review of *Piper* species growing in the Brazilian state of Paraná with emphasize on the vegetative anatomy and biological activities. Bot. Rev..

[37] Bokhari TZ, Younis U, Ummara U, Sharif T, Khan MA (2013). Some parasitic plants of Pakistan—Anatomical and taxonomic attributes. Int. J. Sci. Eng. Res..

[38] Grazi VG, Urech K (1981). Einige morphologische merkmale der mistelheere (*Viscum album* L.) und deren taxonomische bedeutung. Beitr. Biol. Pflanzen..

[39] Heide-Jørgensen, H. S. The Mistletoe *Viscum album* https://viscum.dk/wp-content/uploads/2020/12/Viscum_2015_english_small.pdf (2015).

[40] Pauzer MS (2021). *Eucalyptus cinerea*: microscopic profile, chemical composition of essential oil and its antioxidant, microbiological and cytotoxic activities. Braz. Arch. Biol. Technol..

[41] Formagio ASN (2022). *Palicourea tomentosa* (Aubl.) Borhidi: Microscopy, chemical composition and the analgesic, anti-inflammatory and anti-acetylcholinesterase potential. J. Ethnopharmacol..

[42] Bouropoulos N, Weiner S, Addadi L (2001). Calcium oxalate crystals in tomato and tobacco plants: Morphology and in vitro interactions of crystal-associated macromolecules. Chem. Eur. J..

[43] Oliveira, F., Akisue, G. & Akisue, M. K. *Farmacognosia: Identificação de Drogas Vegetais* (Atheneu, 2014).

[44] Furr M, Mahlberg PG (1981). Histochemical analyses of laticifers and glandular trichomes in *Cannabis sativa*. J. Nat. Prod..

[45] Yoder LR, Mahlberg PG (1976). Reactions of alkaloid and histochemical indicators in laticifers and specialized parenchyma cells of *Catharanthus roseus* (Apocynaceae). Am. J. Bot..

[46] Gabe, M. *Techniques Histologiques* (Masson & Cie, 1968).

[47] Johansen, D.A. *Plant Microtechnique* (Mc Graw Hill Book, 1940).

[48] Mace ME, Howell CR (1974). Histochemistry and identification of condensed tannin precursor in roots of cotton seedlings. Can. J. Bot..

[49] Pearse, A. G. E. *Histochemistry: Theoretical and Applied* (The Williams & Wilkins Company, 1972).

[50] Cain AJ (1947). The use of Nile blue in the examination of lipids. J. Cell Sci..

[51] O’Brien, T. P. & McCully, M. E. *The Study of Plant Structure: Principles and Selected Methods* (Termarcarphi Pty Ltd, 1981).

[52] Vidal BC (1970). Dichroism in collagen bundles stained with Xylidine-Ponceau 2R. Annales d’Histochimie..

[53] Fisher DB (1968). Protein staining of ribboned epon sections for light microscopy. Histochemie..

[54] Dunnigan MG (1968). The use of nile blue sulphate in the histochemical identification of phospholipids. Stain Technol..

[55] Gardner RO (1975). Vanillin-hydrochloric acid as a histochemical test for tannin. Stain Technol..

[56] Pérez-de-Luque A (2006). Mucilage production during the incompatible interaction between *Orobanche crenata* and *Vicia sativa*. J. Exp. Bot..

[57] Azuma JI, Kim NH, Heux L, Vuong R, Chanzy H (2000). The cellulose system in viscin from mistletoe berries. Cellulose.

[58] O’Brien TP, Feder N, McCully ME (1964). Polychromatic staining of plant cell walls by toluidine blue O. Protoplasma.

[59] Kraus JE (1998). Astra blue and basic fuchsin double staining of plant materials. Biotech. Histochem..

[60] Berlyn, G. P. & Miksche, J. P. *Botanical Microtechnique and Cytochemistry* (ed. Sass J. E.) 1–326 (Iowa State University Press, 1976).

[61] Fuchs CH (1963). Fuchsin staining with NaOH clearing for lignified elements of whole plants or plants organs. Stain Technol..

